# Investigation of pathology, expression and proteomic profiles in human *TREM2* variant postmortem brains with and without Alzheimer’s disease

**DOI:** 10.1111/bpa.12842

**Published:** 2020-04-29

**Authors:** Christina E. Toomey, Wendy Heywood, Bridget C. Benson, Georgia Packham, Kevin Mills, Tammaryn Lashley

**Affiliations:** ^1^ The Queen Square Brain Bank for Neurological Disorders Department of Clinical and Movement Neuroscience UCL Queen Square Institute of Neurology London UK; ^2^ Department of Neurodegenerative diseases UCL Queen Square Institute of Neurology London UK; ^3^ Centre for Translational Omics Great Ormond Street Institute of Child Health UCL London UK

**Keywords:** Alzheimer’s disease, amyloid, APOE, microglia, multiomics, neuroinflammation, tau, TREM2

## Abstract

Triggering receptor expressed on myeloid cells 2 *TREM2* was identified as a risk factor for late onset Alzheimer’s disease (AD). Here we compared *TREM2* cases with a variant (*TREM2^+^)* and cases without a *TREM2* variant (*TREM2^−^*), considering pathological burden, inflammatory response and altered canonical pathways and biochemical functions between the cohorts. We hypothesised that *TREM2^+^* cases would have a loss of function, indicating an altered inflammatory profile compared to *TREM2^−^* cases. Immunohistochemistry was performed using antibodies against Aβ, tau and microglia markers in *TREM2^+^* cases, with and without AD, which were compared to sporadic *TREM2^−^* AD, familial AD and neurologically normal control cases. Aβ and tau load were measured along with the composition of Aβ plaques, in addition to microglial load and circularity. Expression and proteomic profiles were determined from the frontal cortex of selected cases. *TREM2^+^* control cases had no Aβ or tau deposition. No differences in the amount of Aβ or tau, or the composition of Aβ plaques were observed between *TREM2^+^* and *TREM2^−^* SAD cases. There were no differences in microglial load observed between disease groups. However, the *TREM2^+^* SAD cases showed more amoeboid microglia than the *TREM2^−^* SAD cases, although no differences in the spatial relationship of microglia and Aβ plaques were identified. Visualisation of the canonical pathways and biological functions showed differences between the disease groups and the normal controls, clearly showing a number of pathways upregulated in *TREM2^+^* SAD, *TREM2^−^* SAD and FAD groups whilst, the *TREM2^+^* controls cases showed a downregulation of the majority of the represented pathways. These findings suggest that the *TREM2^+^* control group, although carrying the *TREM2^+^* variant, have no pathological hallmarks of AD, have altered microglial and expression profiles compared to the *TREM2^+^* SAD cases. This indicates that other unknown factors may initiate the onset of AD, with *TREM2* influencing the microglial involvement in disease pathogenesis.

## Introduction

Triggering receptor expressed on myeloid cells 2 (TREM2) was identified as a genetic risk factor for late onset Alzheimer’s Disease (AD) with a similar odds ratio to *APOE ε4* in 2012, although varying odds ratios have been found in different populations (1, 16, 18, 25, 28, 33, 43, 49, 52). The frequency of the *R47H* variant is 0.23‐0.25 depending on different reports with an estimated odds ratio of 4.46 for AD which increases to 4.62 in cases with a European descent (2). TREM2 is a 40kD, 230 amino acid transmembrane protein belonging to the immunoglobulin family that is expressed on the plasma membrane of a number of different dendritic cells, including microglia (24, 27, 42, 44). It is highly conserved and is thought to be a hub or highly connected gene for microglia in a number of different brain regions, including regions that are affected in AD (13, 35). TREM2 is thought to function through two different pathways, one suppressing inflammation and one that aids phagocytosis of any debris in or around neurons (14, 16, 45, 56). Specifically inhibiting the release and secretion of microglial cytokines and releasing tumor necrosis factor (TNF) to promote survival (21). Multiple studies have shown that when *TREM2* is downregulated there is less microglial activation, impaired phagocytosis of injured neurons/Aβ plaques and an increase in levels of TNF and nitric oxygen species whereas when *TREM2* is overexpressed there is increased phagocytosis and a decrease in pro‐inflammatory signals (24, 54).

The role of TREM2 in AD has been investigated in animal models with amyloid or tau pathology and was found to be upregulated or positively correlated with the presence of amyloid plaques and phosphorylated tau (14, 16, 21, 32, 34, 35, 38, 57). However, Lue *et al* (32) showed no correlation between TREM2 levels and amyloid plaque burden but did confirm the correlation with tau pathology in human postmortem temporal cortices from AD cases. However, when TREM2 is upregulated the number of microglia surrounding plaques increases or the activation of inflammatory pathways is triggered via activation of tau kinases, leading to greater levels of phosphorylated tau (20, 21, 38, 61).

Numerous studies have implicated TREM2 in Aβ phagocytosis by increasing activation or proliferation of microglia around the amyloid plaques. It has been proposed that a microglia barrier expressing TREM2 restricts amyloid plaque growth protecting neurons from damage (61). Jay *et al*, demonstrated that in *APPPS1‐21; TREM2^−/−^* models at 4 months of age there was reduced Aβ in the hippocampus but no change in the cortical load compared to *APPPS1‐21; TREM2^+/+^* mice and at 8 months of age there was no difference in amyloid load measured in the hippocampus (20). Moreover, 8.5 months of age *5xFAD; TREM^−/−^* mice showed no effect on the Aβ cortical load, but had an increase in hippocampal Aβ deposition (58, 59). In addition, when *TREM2* was overexpressed in primary microglia from the same mice at 7 months and 18 months, there was increased Aβ phagocytosis or no response from the microglia, respectively (21). However, the 18‐month‐old microglia were less able to phagocytose Aβ than the 7‐month‐old microglia in normal conditions. The effect of TREM2 on tau deposition has been investigated in P301S mice, showing increased tau pathology and phosphorylation. Whereas overexpression of *TREM2* at 7 months rescued this effect by decreased phosphorylation and reduced neuronal and synaptic loss in the hippocampus (21, 22).

Variance in pathology observed using mouse models has led to speculation about the effect TREM2 is playing on inflammatory processes when the *TREM2 R47H* variant is present. TREM2 in mice has been shown to act upon different mechanisms to human *TREM2* variants. The *R47H* variant reduces *TREM2* mRNA expression and splicing but the normal transcripts were observed in human *TREM2 R47H* (60). Additionally, *TREM2 R47H* and *R62H* variants in humans were shown to have a decreased reactive microglial phenotype compared with the TREM2‐dependent mechanisms seen in mice models using single‐nucleus transcriptomics (63). These studies highlight the differences observed between mice and humans and show the need for further studies using *TREM2^+^* variant human post mortem tissue.

*APOE* was identified as a genetic risk factor for AD, with different allelic compositions determining the level of risk (50). As both *APOE ε4* and *TREM2* have been identified as genetic risk factors for AD, and TREM2 has been shown to also bind anionic lipids, it has been hypothesised that TREM2 functions along similar mechanisms as APOE (9, 58). TREM2 is able to bind to APOE and APOE has been reported to be upregulated in microglia that surround amyloid plaques. The microglia that surround the neuritic plaques in AD were found to have a distinct phenotype to other microglial cells and have been termed “dark microglia,” disease‐associated microglia (DAM) or microglia of neurodegeneration (MGnD) by different groups (3, 26, 29). These microglia have been found to act differently to other activated microglia through the upregulation of several genes that stimulate the TREM2 pathway, including APOE (26).

Investigations have been undertaken on postmortem human brain samples (29, 46) carrying various *TREM2* variants that have confirmed findings shown in mouse models. These included a reduction in plaque‐associated microglia, with no overall reduction in the number of microglia (29, 46), and an increase in neuritic plaque burden and overall tau burden in the hippocampus of *TREM2* variant cases (46). Here we extend the investigations in human postmortem brains of sporadic AD cases with a *TREM2* variant (*TREM2^+^* SAD), sporadic AD cases without a *TREM2* variant (*TREM2^−^* SAD), familial AD cases (FAD), normal controls with a *TREM2* variant (*TREM2^+^* controls) and normal controls without a *TREM2* variant (controls). We hypothesised that *TREM2^+^* cases would have a loss of function, indicating an altered inflammatory profile compared to *TREM2^−^* cases. Through immunohistochemical techniques, nanostring technology and label‐free quantitative mass spectrometry we show that whilst Aβ plaques and neurofibrillary tau tangles (NFT’s) do not significantly change between groups, the microglial phenotype is altered alongside differences in genetic and proteomic profiles between the *TREM2^−^* SAD and *TREM2^+^* SAD cases and between the *TREM2^+^* SAD and *TREM2^+^* controls.

## Material and Methods

### Cases

All cases were obtained through the brain donation program at Queen Square Brain Bank for Neurological Disorders (QSBB). Standard diagnostic criteria were used to assess AD pathology in all cases (5, 39, 55). *TREM2^+^* SAD cases (n = 3, 2 R47H, 1 D87N), *TREM2^+^* control cases (n = 2, 2 R47H), *TREM2^−^* SAD cases (n = 19), FAD cases (n = 11) and control cases (n = 6) were used for this study. The demographic data for all cases is shown in Table 1. Cases were matched for age of onset and age at death where possible. There was no significant difference in age of onset between *TREM2^+^* SAD cases and *TREM2^−^* SAD cases (*P* = 0.6769) or in age at death between *TREM2^+^* SAD cases and *TREM2^−^* SAD cases (*P* > 0.9999). All *TREM2* variant cases used in this study were genotyped as part of the study discovering *TREM2* as a risk variant (16). Ethical approval for the study was obtained from the Local Research Ethics Committee of the National Hospital for Neurology and Neurosurgery.

**Table 1:** Demographics of cases used in the study. Including gender, age at onset and age at death. Known mutations are listed for the FAD cases and *TREM2^+^* SAD cases. APOE status was determined for all cases where frozen tissue was available for DNA extraction. Pathological diagnosis including Braak and Braak stage, Thal Phase, CERAD score and “ABC” score are documented.

Case	Gender	Age of onset		Age at death	Disease duration		Postmortem delay (h)	Mutations	ApoE status	Clinical diagnosis	Pathological diagnosis	Braak and Braak	Thal	CERAD	ABC
*TREM2^+^ SAD cases*															
1	M		55	64	9	35:40:00		R47H	E3/E4	CBS	AD	6	5	Frequent	A3B3C3
2	F		56	66	15	51:20:00		R47H	E4/E4	SAD	AD	6	5	Frequent	A3B3C3
3	M		60	71	11	52:30:00		D87N	E3/E4	FTD	AD	6	5	Frequent	A3B3C3
4	F		36	41	5	64:15:00		R47H Pre 200 PS1	E3/E3	SAD	AD	6	5	Frequent	A3B3C3
*TREM2^+^ control cases*															
5	M		–	76	–	60:35:00		R47H	E2/E2	Control	Control	0	0	None	A0B0C0
6	M		–	82	–	25:30:00		R47H	E3/E3	Control	Control	0	0	None	A0B0C0
*TREM2^−^ SAD cases*															
7	M		63	73	10	31:10:00		–	–	SAD	AD	6	5	Frequent	A3B3C3
8	F		51	63	12	16:00:00		–	E3/E4	SAD	AD	6	5	Frequent	A3B3C3
9	F		51	62	11	62:55:00		–	E3/E4	SAD	AD	6	5	Frequent	A3B3C3
10	F		65	70	5	46:58:00		–	E3/E3	SAD	AD	5	5	Moderate	A3B3C3
11	M		64	77	13	90:05:00		–	E4/E4	SAD	AD	6	5	Frequent	A3B3C3
12	F		49	62	13	76:40:00		–	E3/E3	SAD	AD	6	5	Frequent	A3B3C3
13	M		72	88	16	85:35:00		–	E3/E4	SAD	AD	6	5	Frequent	A3B3C3
14	M		52	69	17	35:04:00		–	E3/E3	SAD	AD	6	5	Frequent	A3B3C3
15	M		65	72	7	38:55:00		–	E3/E4	SAD	AD	5	5	Moderate	A3B3C3
16	F		76	85	9	90:20:00		–	E3/E4	SAD	AD	6	5	Frequent	A3B3C3
17	M		55	64	9	76:45:00		–	E3/E4	SAD	AD	6	5	Frequent	A3B3C3
18	F		69	74	5	93:40:00		–	–	SAD	AD	6	5	Frequent	A3B3C3
19	M		80	85	5	129:15:00		–	–	SAD	AD	5	5	Moderate	A3B3C3
20	F		46	52	6	51:55:00		–	–	LBD	AD	6	5	Frequent	A3B3C3
21	F		49	55	6	47:50:00		–	E3/E3	SAD	AD	6	5	Frequent	A3B3C3
22	M		67	72	5	91:10:00		–	E2/E4	bvFTD	AD	6	5	Frequent	A3B3C3
23	F		65	79	14	22:30:00		–	E3/E4	SAD	AD	6	5	Frequent	A3B3C3
24	M		52	68	16	35:20:00		–	E3/E4	FTD/Picks	AD	6	5	Frequent	A3B3C3
25	M		58	68	10	52:05:00		–	E3/E4	SAD	AD	6	5	Frequent	A3B3C3
*FAD cases*															
26	F		48	59	11	26:15:00		PSEN1 202F	E4/E4	FAD	AD	6	5	Frequent	A3B3C3
27	F		35	52	17	32:30:00		PSEN1 Intron 4	E4/E4	FAD	AD	6	5	Frequent	A3B3C3
28	M		61	70	9	161:15:00		PSEN1 S132A	E3/E4	FAD	AD	5	5	Frequent	A3B3C3
29	M		42	51	9	43:10:00		PSEN1 mutation	E3/E3	FAD	AD	6	5	Frequent	A3B3C3
30	F		48	59	11	89:42:00		V717L APP	E3/E3	FAD	AD	6	5	Frequent	A3B3C3
31	M		60	66	6	68:05:00		V717L APP	E3/E3	FAD	AD	6	5	Frequent	A3B3C3
32	M		42	47	5	43:50:00		PSEN1 A434T & T291A	E3/E3	MSA	AD	5	5	Frequent	A3B3C3
33	F		46	66	20	31:55:00		R278I	E3/E4	FAD	AD	6	5	Frequent	A3B3C3
34	F		33	37	4	24:15:00		E120K exon 5 PSEN1	E3/E3	FAD	AD	6	5	Frequent	A3B3C3
35	F		44	56	12	16:25:00		APP V717I	E3/E3	FAD	AD	6	5	Frequent	A3B3C3
36	F		39	47	8	–		PSEN1 Intron 4	E3/E3	FAD	AD	6	5	Frequent	A3B3C3
*Control cases*															
37	M		–	87	–	57:00:00		–	E3/E3	Normal	Normal	2	3	Moderate	A2B1C2
38	M		–	81	–	50:55:00		–	E2/E2	Normal	Normal	2	1	Sparse	A1B1C0
39	F		–	73	–	24:00:00		–	E3/E4	Normal	Normal	2	2	Sparse	A1B1C2
40	M		–	88	–	16:15:00		–	E3/E3	Normal	Normal	2	2	Moderate	A1B1C2
41	F		–	80	–	49:10:00		–	E3/E3	Normal	Normal	0	2	None	A0B1C0
42	F		–	93	–	29:40:00		–	E3/E3	Normal	Normal	3	1	Moderate	A1B2C2
*Averages*															
TREM2	2F:4M		52	67	10	50:35:00		–	–	–	–	4	3	–	A2B2C2
SAD	9F:10M		60	70	10	61:48:00		–	–	–	–	6	5	–	A3B3C3
FAD	7F:4M		45	55	10	53:44:12		–	–	–	–	6	5	–	A3B3C3
Control	3F:3M		–	84	–	37:50:00		–	–	–	–	2	2	–	A1B1C1

### APOE genotyping

#### DNA extraction

About 100 mg of frozen cerebellum from all cases, except cases 7, 18‐20 where no frozen tissue was available was homogenised in extraction buffer (0.1 M NaCl, 20 mM Trizma base, 25 mM EDTA disodium, 0.5% SDS) and proteinase‐K solution (10 mg/mL) and samples digested at 55°C. A 1:1:1 mix of phenol, chloroform and IAA added and centrifuged for 5 minutes at 12 000 rpm. The aqueous layer was removed and 3M NaAC pH5.3 added. 100% ethanol was added to precipitate the DNA. The pellets were dried at room temperature and resuspended in TE (Tris‐EDTA) solution and stored at 4°C.

#### Genotyping

The Qiagen PCR Mix–GC Rich kit was used. The primers used to determine the ApoE status were previously reported (10). The master mix was added to the DNA and a PCR run with the following settings: 94°C for 5 minutes, 30× (94°C for 30 s, 60°C for 30 s, 72°C for 30 s), 72°C for 5 minutes before being left at 4°C. A 3% metaphor agarose gel/ 2% normal agarose was prepared with GelRed dye added. The digested PCR end product for each sample was added to the gel, which was run for 1 h 30 minutes at 80 V before being visualised in a DNR Bio‐Imaging Systems MiniBIS Pro.

### Immunohistochemistry

Eight‐micron‐thick formalin‐fixed paraffin‐embedded (FFPE) tissue sections from the frontal cortex, temporal cortex and hippocampus were cut from the cases listed in Table 1. Sections were deparaffinised in xylene and rehydrated using graded alcohols. Immunohistochemistry for all antibodies required pressure cooker pre‐treatment for 10 minutes in citrate buffer pH 6.0. Aβ immunohistochemistry also required formic acid pre‐treatment prior to pressure cooking. Endogenous peroxidase activity was blocked in 0.3% H_2_O_2_ in methanol for 10 minutes and non‐specific binding blocked with 10% dried milk solution. Tissue sections were incubated with primary antibodies; Aβ (1:100; Dako); AT8 (tau, 1:600; Thermo); Iba1 (microglial, 1:1000; Wako); CD68 (microglial, 1:100, Dako); CR3‐43 (microglial, 1:150, Dako); P2RY12 (microglial, 1:100; Sigma); Glial fibrillary acidic protein (GFAP) (astrocytic, 1:1000 Dako) for 1 h at RT, followed by biotinylated anti‐rabbit IgG (1:200; Dako) or biotinylated anti‐mouse IgG (1:200; Dako) for 30 minutes at RT and Avidin‐Biotin complex (30 minutes; Dako). Colour was developed with di‐aminobenzidine/H_2_0_2_ (30). Stained sections were digitised using a Leica SCN400F slide scanner.

### Double immunohistochemistry and Thioflavin‐S staining and analysis

Formalin‐fixed paraffin‐embedded tissue sections were cut from the frontal cortex of three *TREM2^+^* SAD cases (cases 1‐3) and three *TREM2^−^* SAD cases (cases 13,15,17). Sections underwent immunohistochemistry as described above for: ionized calcium binding adapter molecule 1 (Iba1), CD68 and CR3‐43 using Tyramide Signal Amplification kit as the chromogen. Thioflavin‐S was applied for 7 minutes and differentiated with 70% ethanol. Slides were visualised under a Leica DM5500 fluorescent microscope. Z‐stack images at 63× magnification were collected from the frontal grey matter and antibody stain to analyse the microglial load observed in diffuse and dense core amyloid plaques. Using Image J, the channels were separated, the total plaque area calculated together with the percentage area positive for microglia markers. The sum of percentages for all 20 diffuse and all 20 dense core plaques were then taken for each case.

### Pathological analysis

#### Digital morphological analysis

Digital images for Aβ, tau and microglial markers immunohistochemistry were viewed using Aperio Imagescope (v12.3.0.5056). We analysed the areal fraction of the immunohistochemical staining (all antibodies), as well as the number of stained microglia and the circularity of the microglia. Using Image J software (https://imagej.nih.gov/ij/) and a python script 10 randomised snapshots, representing 500 µm^2^, were generated from the extracted regions of interest. The region of interest included all six cortical layers of the cortex and all areas of the hippocampus. Bland‐Altman plots were performed to determine the reliability of the method and how many snapshots were needed. The 10 snapshots were then used to determine the areal fraction for each immunohistochemical preparation. Areal fractions were calculated for each snapshot and means were taken for each case, each region and each antibody. In the microglial stained preparations the number and the circularity of microglia were also determined. A score closer to one indicated the microglia were more amoeboid in shape whereas a score closer to 0 indicates the microglia were more ramified. An average circularity value was taken for each snapshot and a mean of the snapshots were taken for each case, region and stain. Kruskal‐Wallis one‐way ANOVA tests with Dunn’s multiple comparisons were performed to determine any significant differences for all types of analysis at a level of *P* < 0.05.

#### Manual analysis

The digital morphological analysis would not allow distinction between different Aβ plaque types, this analysis required manual counting. Using the randomized snapshots generated from the digital morphological analysis, different plaque types (diffuse or dense‐core) were manually counted to determine how many plaques were in the representative sample. Dense core plaques were only counted when a dense core could be visualised, otherwise they were counted as diffuse plaques.

### Expression analysis

#### RNA extraction

RNA extraction was performed using the Qiagen RNeasy kit and protocol from 100 mg of frozen frontal cortex following the manufacturer’s instructions. The RNA concentration and purity was measured using an Eppendorf spectrophotometer.

#### Nanostring

All RNA samples were analysed on the NanoString Human Inflammation panel containing 256 genes and 30 extra genes relevant to AD. Excess probes were removed and probe/target complexes aligned and immobilised in the nCounter cartridges. Raw data was analysed on nSolver software (15). The results were normalised using positive controls and five housekeeping genes (*CLTC, GAPDH, GUSB, PGK1 and TUBB*). All pairwise ratios between groups were made from the normalised data and two‐tailed t‐tests were performed to establish any significance at *P* < 0.05.

### Mass spectrometry

Frozen frontal cortex samples were homogenised in 50 mM Ambic buffer with 2% ASB‐14 and pooled per disease group (three cases per pooled sample other than *TREM2^+^* controls that only had 2). Proteins were extracted into two fractions; soluble supernatant and the insoluble pellet fraction. For each fraction Label‐free mass spectrometry was performed with a SYNAPT G2‐Si High Definition mass spectrometer (Waters, UK) with 2D fractionation as previously described (8, 40). There were four fractions run for each sample and 0.5 µg of protein were injected per fraction per run. The raw data were imported into Progenesis for proteomics software (Nonlinear dynamics, UK) and processed. Identifications were obtained by searching the data against the human reference proteome (2016). Data for identifications with more than 1 unique peptide were exported for downstream analysis.

### Bioinformatics

As a label‐free approach was taken, all genes or proteins that met the threshold set (*P* < 0.05 for nanostring, >1.5‐fold change compared to controls in expression for proteomics) were put into publicly available databases to assess the relationships between them and the biological processes, molecular functions and cell components that were enriched in the different disease groups. To assess the enriched gene ontology terms Webgestalt (62) was used. GOview was used to compare terms that were over‐represented between regions or disease group. Ingenuity Pathway Analysis software was used to perform in depth canonical pathway analysis.

## Results

### Pathological diagnosis of *TREM2^+^* cases

**Figure 1:**
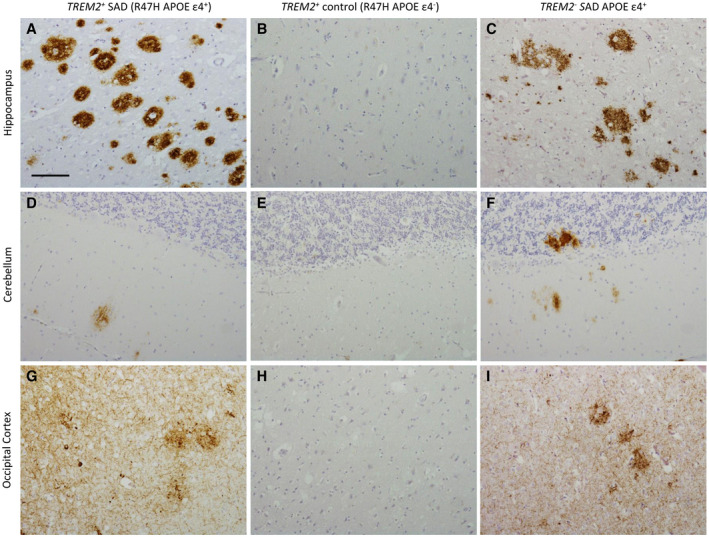
Aβ and tau immunohistochemistry in *TREM2^+^* SAD, *TREM2^+^* control and *TREM2^−^* SAD cases. Aβ immunohistochemistry was carried out to confirm the diagnosis of AD. Aβ plaques were observed in the hippocampus and cerebellum in the *TREM2^+^* SAD cases (**A** and **D**) and *TREM2^−^* SAD cases (**C **and **F**). Whereas the *TREM2^+^* control cases showed no Aβ deposition (**B** and **E**). Tau immunohistochemistry showed severe neuropil threads, neurofibrillary tangles and neuritic plaques in the occipital cortex in the *TREM2^+^* SAD cases (**G**) and *TREM2^−^* SAD cases (**I**). However, no tau positivity was observed for the *TREM2^+^* control cases (**H**). Bar in a represents 100 µm in all panels.

All cases underwent a routine diagnostic assessment. Routine immunohistochemistry was performed on each case to determine the final diagnosis (Table 1). Microscopic observations for all *TREM2^+^* SAD cases (Table 1, cases 1‐3) showed cases reached end stage AD with a score of A3B3C3 according to current diagnostic criteria. Figure 1 demonstrating the presence of Aβ and tau in the hippocampus, cerebellum and occipital cortex, brain regions examined in the diagnostic criteria. Two cases (case 1 and 3) also had additional alpha‐synuclein pathology with Lewy bodies observed in the amygdala and substantia nigra. The two *TREM2^+^* control cases (Table 1, cases 5 and 6) had no Aβ plaque pathology or tau pathology and were diagnosed as neurologically normal controls (Figure 1).

### APOE status and pathological diagnosis

The *APOE* genotypes of cases used in this study are shown in Table 1. Table 2 shows the distribution of *APOE* alleles for all disease groups studied. There were predominantly more *ɛ4* alleles present throughout the cases confirmed with AD pathology. The *TREM2^+^* control cases did not carry any *ɛ4* alleles. However, the *TREM2^+^* SAD cases had at least one *ɛ4* allele.

**Table 2:** The distribution of APOE alleles across case cohorts

Disease group	APOE genotype					
	2/2	2/3	2/4	3/3	3/4	4/4
Control	Y			Y	Y	
TREM2^−^ SAD			Y	Y	Y	Y
FAD				Y	Y	Y
TREM2^+^ Control	Y			Y		
TREM2^+^ SAD					Y	Y

### No difference in amount and type of Aβ plaques in *TREM2^+^* and *TREM2^−^* SAD cases

**Figure 2:**
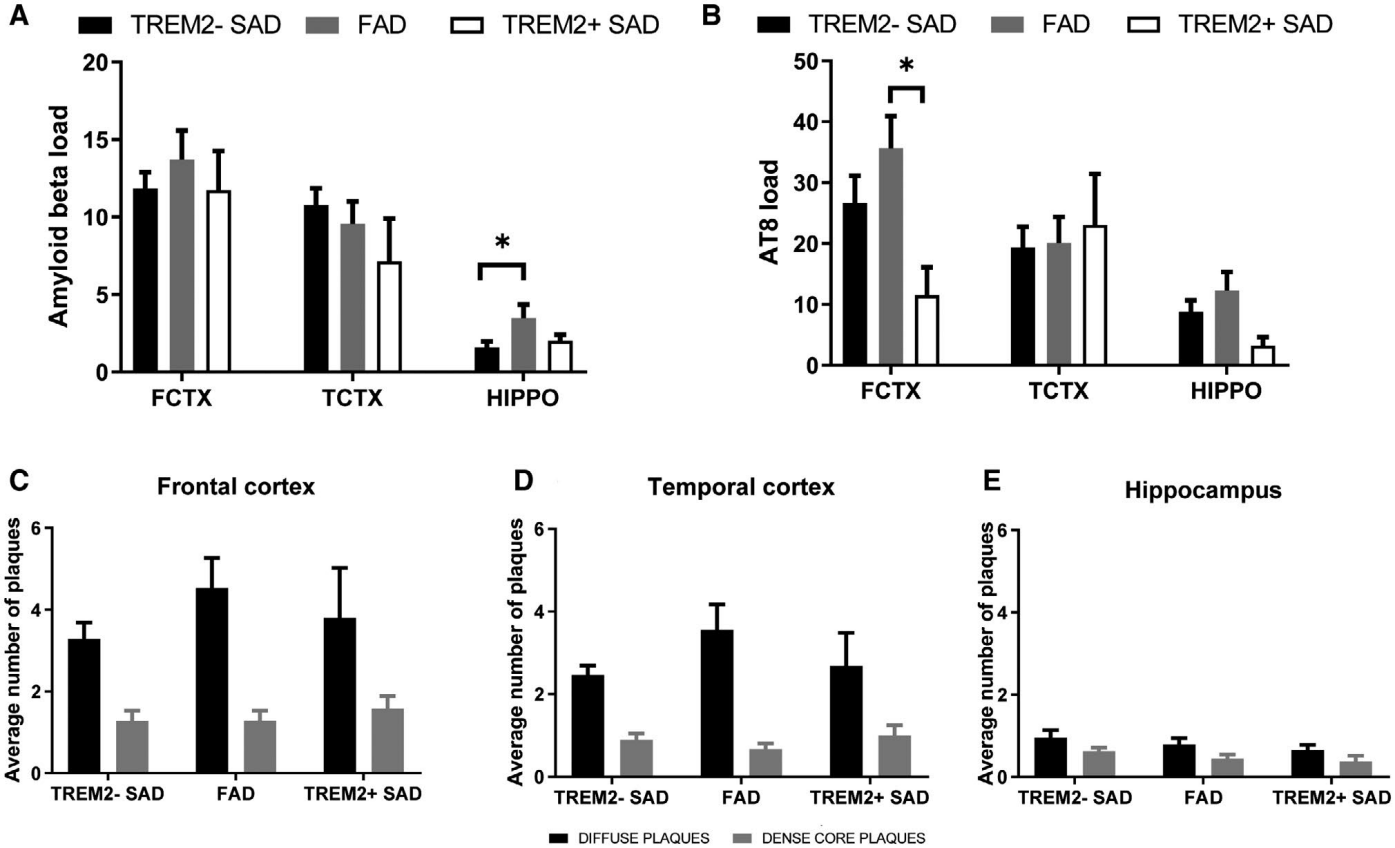
Quantitative analysis of Aβ and tau load and semi‐quantitative analysis of Aβ plaque type. The Aβ load was measured in the *TREM2^+^* SAD, *TREM2^−^* SAD and FAD cases (**A**). Load was measured as mean % area stained in the frontal cortex (FCTX), temporal cortex (TCTX) and hippocampus (HIPPO). The numbers of diffuse or dense‐core plaques were determined in the same cohorts, in the frontal cortex (**C**), temporal cortex (**D**) and hippocampus (**E**). The tau load was determined in the cohorts (**B**). Load measured as mean % area stained. Kruskal‐Wallis one way ANOVA was performed for each region, Wilcoxon‐paired ranks test was performed to determine statistical differences between types of plaques for each disease group in each region. Significance is shown as *P < 0.05.

Aβ load and types of plaques were determined in the different disease groups across three brain regions (Figure 2). There were no significant differences in Aβ load between *TREM2^−^* SAD and *TREM2^+^* SAD cases (*P* = 0.6667). The FAD cases had significantly more Aβ in the hippocampus than both *TREM2^−^* SAD (hippocampus *P* = 0.0411). The Aβ load in the *TREM2^+^* SAD cases mirrored the *TREM2^−^* SAD cases across all regions. Semi‐quantitative assessment was carried out to determine whether different plaque types were more or less prevalent in the *TREM2^+^* SAD cases in the three brain regions. There was no significant difference in the numbers of dense cored plaques or diffuse plaques between the disease groups (*TREM2^+^* SAD, *TREM2^−^* SAD and FAD) for all regions (Figure 2).

### No difference in tau load by immunohistochemical analysis in *TREM2^+^* and *TREM2^−^* SAD cases

The level of tau accumulation was quantified in the three brain regions in *TREM2^+^* SAD cases compared to *TREM2^−^* SAD and FAD cases (Figure 2). There was no significant difference in tau load between *TREM2^+^* SAD cases and *TREM2^−^* SAD cases (*P* = 0.8068). However, there was a significant increase in tau load in the frontal cortex of FAD cases compared to *TREM2^+^* SAD cases (*P* = 0.0248).

### Differences in microglia load and morphology in *TREM2^+^* and *TREM2^−^* cases

**Figure 3:**
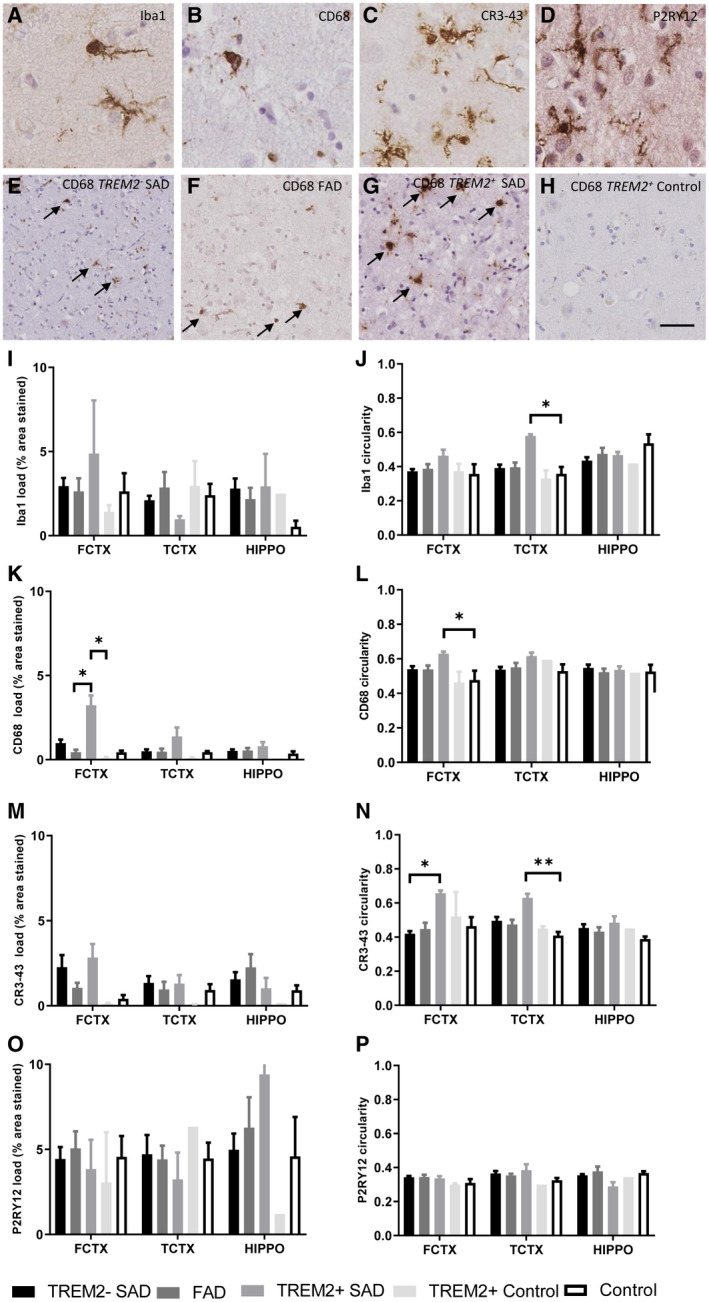
Microglial load and circularity in *TREM2^+^* and *TREM2^−^* cases. Measured using the microglial markers Iba1, CD68, CR3‐43 and P2RY12 in frontal cortex (FCTX), temporal cortex (TCTX) and hippocampus (HIPPO) between *TREM2^+^* SAD, *TREM2^−^* SAD, FAD, *TREM2^+^* controls and normal controls. Representative images of microglial immunohistochemistry for Iba1 (**A**), CD68 (**B**), CR3‐43 (**C**) and P2RY12 (**D**) in case 7. Representative images of CD68 staining in the frontal cortex of a *TREM2^−^* SAD (**E**, case 13), FAD (**F**, case 27), *TREM2^+^* SAD (**G**, case 2) and *TREM2^+^* control (**H**, case 3). Iba1 load (**I**), CR343 load (**J**), CD68 load (**K**), and P2RY12 load (**L**). A measure of circularity was obtained where 1 was a perfect circle whereas a score of 0 was an imperfect shape. Mean values were taken from each case in a disease group. The circularity scores are shown Iba1 circularity (**J**), CD68 circularity (**L**), CR3‐43 circularity (**N**), and P2RY12 circularity (**P**). Black arrows highlight CD68 + ve microglia. Scale bar represents 50 µm. Significance is shown as *P < 0.05, **P < 0.005.

Microglial load was assessed and compared between *TREM2^−^* SAD, *TREM2^+^* SAD, FAD, *TREM^+^* control cases and neurologically normal controls, using four microglial markers across three brain regions (Figure 3A–D). There were no significant differences that could be seen when assessing Iba1, CR343 or P2RY12 load (Figure 3I,M,O). However, *TREM2^+^* SAD cases appeared to have a markedly increased Iba1 load in the frontal cortex and decreased load in the temporal cortex although this did not reach significance. In *TREM2^+^* SAD cases CR343 load appeared to follow the same trend as *TREM2^−^* SAD cases when compared to other disease groups. However, the *TREM2^+^* control cases had significantly less CR343 positive, activated microglia, present compared to the *TREM2^+^* SAD cases. P2RY12 load remained consistent between disease groups although a greater level of variation was observed in the hippocampus compared to other regions, with *TREM2^+^* control cases having markedly less homeostatic microglia than the *TREM2^+^* SAD cases. *TREM2^+^* SAD cases had a significantly higher CD68 load in the frontal cortex compared to the *TREM2^+^* control cases (Figure 3K; *P* = 0.0147) and the FAD cases (Figure 3K; *P* = 0.0127). Representative CD68 images from *TREM2^−^* SAD, *TREM2^+^SAD*, FAD and *TREM2^+^* control cases illustrate these differences (Figure 3E–H).

As microglia have a spectrum of different morphological phenotypes, ranging from a ramified, surveillance phenotype to an amoeboid phagocytic phenotype, the shape of the microglia were also investigated across the different disease groups. This was measured as an index of circularity in which a perfect circle (amoeboid microglia) scored closer to 1 and an imperfect shape (ramified microglia) scored closer to 0. *TREM2^+^* SAD cases had significantly more amoeboid Iba1 microglia than controls in the frontal and temporal cortices (Figure 3J; *P* = 0.0248). *TREM2^+^* SAD cases also had significantly more circular CR343 microglia than the *TREM2^−^* SAD cases (Figure 3N; *P* = 0.0128) in the frontal cortex and more circular microglia than the controls (*P* = 0.0064) in the temporal cortex. Furthermore, *TREM2^+^* SAD cases had significantly more circular CD68 microglia than the controls in the frontal cortex (Figure 3L; *P* = 0.0296). As expected P2RY12 microglia generally had a circularity score closer to 0 than Iba1, CD68 and CR3‐43 microglia (Figure 3P). No major differences were observed between disease groups for P2RY12 circularity.

### No differences in microglial clustering around amyloid plaques in *TREM2^+^* and *TREM2^−^* SAD cases

**Figure 4:**
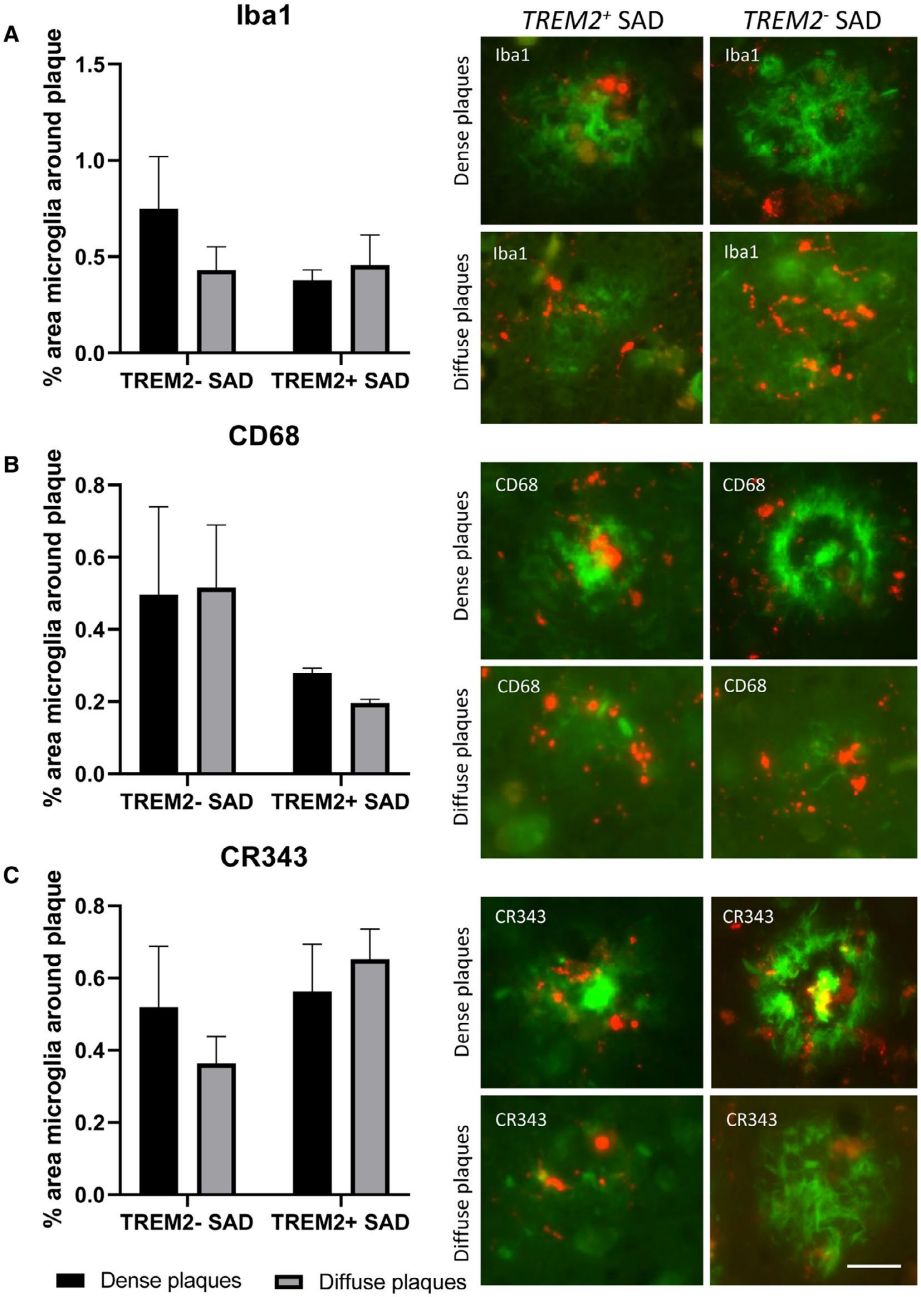
Clustering of microglia around amyloid plaques. Three microglial markers [Iba1 (**A**), CD68 (**B**) and CR3‐43 (**C**)] were used to investigate the area of microglial staining around either dense cored plaques or diffuse plaques. Representative images of dense cored plaques and diffuse plaques with double staining with thioflavin (green) and microglia markers (red). No significant differences were found in the area of microglial staining between *TREM2^−^* SAD and *TREM2^+^* SAD cases. Bar on image represents 25 µm.

The percentage area of microglial staining around diffuse and cored plaques was analysed in *TREM2^−^* and *TREM2^+^* SAD cases to determine if the presence of the *TREM2* variant correlated with the amount of microglial positive staining that clustered around amyloid plaques. Three microglial markers (Iba1, CD68 and CR343) were used to determine differences between different activation states of microglia around the plaques. There were no significant differences between *TREM2^+^* SAD cases and *TREM2^−^* SAD cases in any of the three markers or between diffuse and dense core amyloid plaques (Figure 4). A trend was observed showing *TREM2^+^* SAD cases had less percentage area load of CD68 in both diffuse and dense core plaques than SAD cases; however, this did not reach significance.

### *TREM2* variant effect on astrocyte phenotype

**Figure 5:**
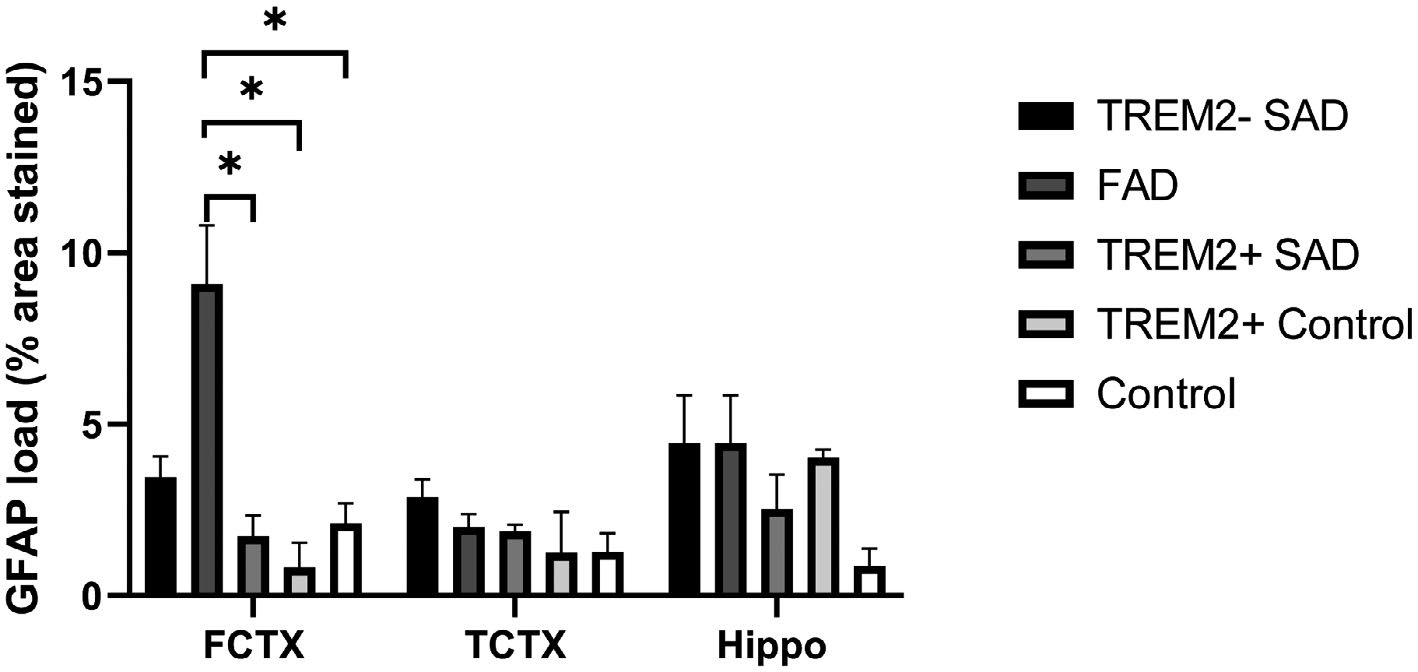
Astrocyte load in *TREM2^+^* and *TREM2^−^* cases. Measured using GFAP in the frontal cortex (FCTX), temporal cortex (TCTX) and hippocampus (HIPPO) between *TREM2^+^* SAD, *TREM2^−^* SAD, FAD, *TREM2^+^* controls and normal controls. No significant differences were observed between *TREM2^+^* and *TREM2^−^* control cases. However, significant differences were observed between FAD and *TREM2^+^* SAD, *TREM2^+^* controls and normal controls. Significance is shown as *P < 0.05.

GFAP is a reactive astrocyte marker. GFAP was found to be upregulated to a higher level in *TREM2^+^* SAD cases at both the gene and protein level. At the gene expression level GFAP was upregulated 1.99‐fold in *TREM2^−^* SAD cases, 2.35‐fold in *TREM2^+^* SAD cases and 1.3‐fold in *TREM2^+^* control cases all compared to neurologically normal controls. At the protein level GFAP was upregulated across all groups but the greatest level of upregulation was observed in the *TREM2^+^* SAD cases in both the soluble and insoluble fractions (3.8‐fold soluble, 3.2‐fold insoluble). As this increase in expression was observed for the *TREM2^+^* SAD cases, we assessed the GFAP pathological load in the frontal cortex, temporal cortex and hippocampal regions (Figure 5). There were no significant differences between *TREM2^+^* SAD cases, *TREM2^−^* SAD cases and *TREM2^+^* control cases. However, there were significant differences between FAD cases and *TREM2^+^* SAD cases.

### Expression analysis of *TREM2^+^* SAD and *TREM2^+^* controls

The NanoString Technologies Human Inflammation panel containing 256 genes and 30 targeted genes specific to AD were processed and analysed on the frontal cortex from all groups and compared with normal controls. Upregulation or downregulation of genes are determined as a ratio to normal controls. A proportion of the genes analysed were upregulated in *TREM2^−^* SAD (124 genes at a significance level of *P* < 0.05, of which 70 genes at *P* < 0.01 and 24 genes at *P* < 0.001). Two genes were downregulated significantly in *TREM2^−^* SAD, MRC1 (macrophage mannose receptor 1) and SNCA (alpha‐synuclein) (*P* < 0.05); in FAD (91 genes were significantly upregulated at level of *P* < 0.05, 22 genes at *P* < 0.01 and 2 genes, NFKB1 (nuclear factor NF‐kappa‐B p105 subunit) and TGFB2 (transforming growth factor beta‐2) at *P* < 0.001). Two genes were downregulated PIK3C2G (Phosphatidylinositol 4‐phosphate 3‐kinase C2 domain‐containing subunit gamma) and SNCA (*P* < 0.05); in *TREM2^+^* SAD cases (22 genes were significantly upregulated at a level of *P* < 0.05); in *TREM2^+^* controls (11 genes were significantly upregulated and 14 genes were downregulated *P* < 0.05) (Supporting Table S1). Visualisation of the canonical pathways and biological functions altered between the disease groups and normal controls highlights the number of pathways predicted to be activated in *TREM2^+^* SAD, *TREM2^−^* SAD and FAD. Moreover, the majority of pathways represented were predicted to be inhibited in the *TREM2^+^* controls cases (Supporting Figure S1). There were several genes that were significantly altered across all disease groups. Genes that demonstrated more than twofold change in expression compared to controls across all three groups included HSPB2 (Heat shock protein beta‐2), TGFB1 (Transforming growth factor beta‐1), CSF1 (Macrophage colony‐stimulating factor 1) and CXCR4 (C‐X‐C chemokine receptor type 4). Several genes showed significantly altered expression in only one disease group. For example, APOE and PEN2 (Gamma‐secretase subunit PEN‐2) were only significantly upregulated in *TREM2^−^* SAD compared to controls but were not significantly altered in any other disease group. Similarly, CD68, IL6R (Interleukin‐6 receptor subunit alpha) and TYROBP (TYRO protein tyrosine kinase‐binding protein) were significantly upregulated in FAD compared to controls. *TREM2^+^* SAD cases were the only group to have a significant upregulation of GRB2 (Growth factor receptor‐bound protein 2).

### Proteomic analysis of *TREM2^+^* SAD and *TREM2^+^* controls

To determine if the genetic expression changes also occur at the translational level, label‐free mass spectrometry was performed to determine changes in protein expression. A total of 6012 proteins were detected in the soluble supernatant fraction and 5571 in the insoluble pellet fraction. Proteins were either detected in both the soluble and insoluble fraction (3269 proteins) or they were unique to the soluble fraction (2743 proteins) or insoluble fraction (2302 proteins). Only proteins that were changing >1.5‐fold compared to normal controls were included (3330 proteins) and both up‐ and downregulation of proteins were observed in every disease group when compared to controls. The top 20 upregulated and downregulated proteins are listed in Table 3. There was very little overlap in these proteins between the different disease groups.

**Table 3:** Top 20 up‐ and downregulated proteins compared to neurologically normal controls across *TREM2^+^* SAD, *TREM2^−^* SAD, FAD and *TREM2^+^* controls cases. Protein ID and the fold change given. Overall top proteins from soluble or insoluble fractions. Fold changes in soluble and insoluble fractions for AD‐related proteins MAPT, APP and APOE compared to normal controls

*TREM2^−^*SAD		FAD		*TREM2* ^+^ SAD		*TREM2* ^+^ Control	
*Upregulated*							
KRT6A	25.32	STOX2	281.43	GOLGA8R	60.79	PSMB7	13.12
KRT6C	8.93	SNRPD3	219.66	APOE	7.77	NEDD8	9.55
DSC1	8.47	TMEM43	152.18	MAPT	7.74	WDR19	9.32
COL25A1	7.94	QRICH2	136.53	ECT2	7.14	SLC14A1	5.92
KRT5	6.57	FUS	131.60	IFIT3	7.11	C2CD3	5.37
KRT16	6.50	CUL5	108.19	C4A	6.83	SHTN1	4.89
CRTC1	6.07	TRPM1	86.62	FHAD1	6.27	PGBD5	4.13
APOE	6.01	PEX19	84.41	SPG7	6.21	WDR47	4.05
KRT17	5.40	SETD5	79.52	PHF5A	5.04	IGHA2	3.88
MAPT	4.49	CDC42BPG	78.53	CPSF7	4.80	LAMA5	3.81
C4A	4.29	PITHD1	73.86	RIC8A	4.58	SCYL1	3.73
IRF2BP1	3.78	RAP1GAP	68.06	ANXA2	4.41	MAP2K1	3.63
OBSCN	3.37	WASL	67.96	STXBP6	4.41	SLC25A5	3.61
QARS	3.35	GLO1	66.47	HIST1H4A	4.23	PSAP	3.50
COL9A2	3.29	DNM1L	65.49	ZBTB38	4.21	NEB	3.50
KRT1	3.13	CUX2	65.09	SQSTM1	4.16	PARP1	3.29
ASAP2	3.10	ATPAF1	56.80	GRIA2	4.09	KRT10	3.27
UBN1	3.08	RHOA	55.35	RBM25	3.98	LAMA2	3.25
ARFGEF1	3.03	GRB2	54.74	APP	3.91	CCDC30	3.16
DCTN2	2.96	UTP15	54.63	PSMC4	3.85	MAP1B	3.14
*Downregulated*							
ASAP3	−7.45	TTC23	−72.67	CCNG1	−18.89	ASAP3	−54.57
VGF	−4.32	INPP4A	−61.52	ASAP3	−12.19	CDH8	−48.09
TBC1D8B	−4.16	SEC14L2	−46.27	GPATCH2	−8.72	WDFY1	−11.28
SCG2	−3.16	TCEB2	−15.76	KSR1	−4.69	COX6A1	−9.54
USP4	−2.91	APCS	−9.98	EPB42	−4.30	SRRM2	−8.96
VPS51	−2.80	TNIK	−8.80	ZEB2	−4.22	AGFG1	−7.18
HIST1H1E	−2.55	PGAM5	−7.15	SRRM2	−3.66	RAB24	−6.49
KPNA6	−2.52	KIAA1468	−7.01	FBF1	−3.53	MKRN1	−6.47
TCEAL3	−2.48	OGDHL	−6.51	ASNS	−3.45	PXN	−6.28
TPM2	−2.47	KTI12	−5.07	SI	−3.27	PANK4	−6.08
FARSA	−2.44	SAMD11	−4.15	CCDC51	−3.10	GC	−5.48
SYNPO	−2.43	LYN	−4.07	TAGLN	−2.89	H3F3A	−5.42
ALDH3A2	−2.40	SDSL	−4.03	NUCKS1	−2.84	HBB	−4.95
IRF2BP2	−2.38	PIP4K2A	−4.02	CIRBP	−2.76	FDFT1	−4.82
TNRC18	−2.35	NPM1	−3.95	HNRNPUL1	−2.69	TECPR1	−4.75
TAGLN	−2.31	HNRNPLL	−3.89	PXN	−2.67	CA1	−4.52
PRIM1	−2.30	HNRNPM	−3.79	C2CD3	−2.65	PTPRE	−4.50
KSR1	−2.29	KLC2	−3.59	EPC2	−2.64	CUX2	−4.43
SENP6	−2.21	IFIT3	−3.59	PITPNM3	−2.60	ERO1A	−4.40
SLC12A7	−2.20	SNRPD1	−3.58	WNK2	−2.60	VARS	−4.28

Gene symbol	Protein name	Fold change compared to control			
		*TREM2* ^−^ SAD	FAD	*TREM2* ^+^ SAD	*TREM2* ^+^ Control
*Soluble fraction*					
APP	Amyloid beta A4 protein	–	1.21	1.06	−1.15
MAPT	Microtubule‐associated protein tau	1.46	2.31	1.04	−1.44
APOE	Apolipoprotein e	−1.05	1.16	1.24	1.12
*Insoluble fraction*					
APP	Amyloid beta A4 protein	2.92	2.83	3.91	−1.34
MAPT	Microtubule‐associated protein tau	4.49	3.76	7.74	−1.24
APOE	Apolipoprotein e	6.01	7.18	7.77	1.16

Ingenuity pathway analysis highlighted that the top canonical pathways (predicted to be activated or inhibited by the expression of proteins represented in them) differ between the soluble and insoluble fractions. The top pathways, represented by the identified proteins in the soluble fraction (Supporting Figure S2) are HIPPO signalling, Melatonin signalling, LXR/RXR activation, amyloid processing, actin cytoskeleton signalling and neuroinflammation signalling. HIPPO signalling, amyloid processing and neuroinflammation signalling are predicted predominantly to be activated across the disease groups, whereas melatonin signalling, LXR/RXR activation and actin cytoskeleton signalling predicted activation and inhibition were more mixed between groups (Supporting Figure S2). The top canonical pathways in the insoluble fraction (Supporting Figure S2) are EIF2 signalling, RhoA signalling, signalling by Rho family GTPases, ERK5 signalling, glioma signalling and ephrin receptor signalling. EIF2 signalling and glioma signalling are predominantly predicted to be inhibited across the disease groups with *TREM2^+^* control cases having the opposite prediction. RhoA signalling, signalling by Rho family GTPases, ERK5 signalling and ephrin receptor signalling have a more activated prediction. The *TREM2^+^* control group has a different pattern showing predominantly predicted activation throughout the top canonical pathways, whereas the other groups are more varied between activation and inhibition.

### Proteomic expression of known AD markers

Several proteins known to be related to AD or neurodegeneration were also altered (Table 3). APP and MAPT are the proteins directly related to AD pathology and in both cases these were upregulated in all groups other than the *TREM2^+^* control cases, in which they were downregulated when compared to normal controls. For APP, 3.9‐fold change was observed in the *TREM2^+^* SAD cases in which it was one of the highest 20 proteins observed to be upregulated. For MAPT, the greatest fold change difference was also observed in the *TREM2^+^* SAD cases (7.7‐fold). It was in the highest 20 proteins upregulated for *TREM2^−^* SAD and *TREM2^+^* SAD. GFAP was upregulated across all groups but the greatest level of upregulation was observed in the *TREM2^+^* SAD group in both the soluble and insoluble fractions (3.8‐fold soluble, 3.2‐fold insoluble). APOE was upregulated across all groups in the insoluble fraction, being in the highest 20 proteins upregulated for all groups other than *TREM2^+^* control cases. Again, the highest level of upregulation was observed in the *TREM2^+^* SAD cases (7.8‐fold, insoluble fraction).

### Multi‐omic comparison

The genetic expression data obtained through nanostring methods and the protein expression data using label‐free mass spectrometry were compared with its limitations in mind. As the nanostring data only investigated 256 neuroinflammatory genes and 30 genes specific to neurodegeneration, neuroinflammatory pathways are more likely to appear as represented when comparing the two datasets. It is, therefore, no surprise that when the top canonical pathways represented in each disease group and using each experiment were compared, the neuroinflammatory signalling pathway was one of the top pathways represented across disease groups. (Supporting Figure S3). However, as this pathway is one of the top canonical pathways represented in the proteomics data, it shows that neuroinflammation is still a major factor at the protein level and should be investigated thoroughly. The neuroinflammation signalling pathway is predicted to be activated in both the nanostring and proteomic data of all groups apart from the *TREM2^+^* control group, in which it is inhibited. When comparing the canonical pathways, the general pattern shows that the pathways are predicted to be activated in the genetic expression data but inhibited in many of the proteomic data. Additionally, the *TREM2* ^+^ control group had a different profile in that the pathways most highly represented in this group are predicted to be inhibited at the genetic level but activated at the protein expression level. This again highlights how different the *TREM2* ^+^ control group are to not only the other AD groups but the other cases that have a *TREM2* variant. This suggests that there is regulation between the transcriptional and translational level that needs further investigation.

## Discussion

We undertook a morphological and biochemical study on a cohort of SAD and control cases found with and without a *TREM2* variant and FAD cases. All *TREM2^+^* SAD cases were diagnosed with AD and all carried an *APOE ε4* allele. The *TREM2^+^* control cases, were negative for an *APOE ε4* allele and had no Aβ plaque or tau pathology. When comparing the pathological burden between *TREM2^+^* and *TREM2^−^* SAD cases we found no differences in the Aβ plaques types, overall Aβ load or tau burden in three different brain regions. Our results are in support of recent studies showing there is no difference in the overall levels of insoluble Aβ between *TREM2^+^* and *TREM2^−^* SAD cases (46) and no difference in the composition of the Aβ plaque types. From an Aβ perceptive, the *TREM2^+^* SAD cases are indistinguishable from *TREM2^−^* SAD cases. Whereas, in mouse models overexpressing *TREM2,* decreases in amyloid plaque deposition were observed, and TREM2 deficient models showed conflicting results on the amount of amyloid load between cortical and hippocampal regions (20, 21, 58, 59). This data agree with recent findings that TREM2 acts differently in mice and humans (60, 63). We found no significant differences in overall tau load between *TREM2^+^* and *TREM2^−^* SAD cases in three different brain regions, which is in contrast to a recent postmortem study (46). It has also been shown in *TREM2* deficient mouse models that tau was increased compared to wild types (22, 23).

We used four markers to investigate the microglial load and microglial morphology, with no difference in the microglial load observed with CR343, Iba1 or P2RY12. Iba1 was used as a pan microglial marker that detects ramified and amoeboid forms of microglia (4, 53). CR343, is a glycoprotein that is part of the major histocompatibility complex class II (MHC II) subgroup and was used to identify reactive/activated microglia (11, 36). P2RY12 was identified as a gene unique to microglia and represents homeostatic microglia (6, 17). This is in keeping with a recent postmortem investigation that showed no difference in the microglial load between high AD and *TREM2* variant cases (46). However, an increase in CD68 positive microglia was seen in the frontal cortex in the *TREM2^+^* SAD cases compared to both the *TREM2^+^* controls and controls. CD68 was used as a phagocytic marker, as it is a lysosomal protein found within microglia, monocytes and macrophages (4). Although we found no difference in Iba1 and CR343 load between cases, we observed that the Iba1 positive microglia were more amoeboid in the *TREM2^+^* SAD cases than controls in the frontal and temporal cortices. Whereas the CR343 positive microglia were more amoeboid in the *TREM2^+^* SAD cases than the *TREM2^−^* SAD and FAD cases. We investigated the spatial relationship of the microglia around both dense and diffuse plaques and found that with three microglia markers (Iba1, CD68 and CR343) there were no differences in the amount of microglial staining around the plaques in any of the disease groups. However, the *TREM2^+^* control cases had significantly less CR343 positive, activated microglia, present compared to the *TREM2^+^* SAD cases. Microglia in AD are thought to be responsible for clearing Aβ plaques via phagocytic mechanisms and *TREM2* is thought to have a role in these mechanisms (4, 16, 25). Our data would suggest that the microglia in the *TREM2^+^* SAD cases are capable of reacting and forming amoeboid microglia. However, our results are not able to show whether the microglia are actually capable of phagocytosing the Aβ plaques. Mazaheri et al (2017) showed that *TREM2* deficient microglia have reduced chemotaxis and response to neuronal injury. Alterations in the phagocytic mechanisms in these *TREM2* variant microglia may play a part in their reduced response to neuronal injury.

To decipher the morphology and distribution of TREM2 within the brain and its relationship to microglia and Aβ plaques, we attempted to perform TREM2 immunohistochemistry. Seven different commercial TREM2 antibodies were tried in order to distinguish the level of pathological load of TREM2 in the *TREM2^+^* variant cases compared to *TREM2^−^* cases (data not shown). To identify if these were specific we used double immunofluorescence with microglial markers and found no co‐localisation between TREM2 and the microglial markers. Therefore, we were not convinced at the specificity of the TREM2 antibodies. There have been some studies that report to show human TREM2 immunohistochemistry but with varying results (12, 51). Most recently Raha‐Chowdhury et al (47, 48) have shown some immunohistochemistry with TREM2 in human tissue but it is unknown how well this was characterised. There has been some debate in the field when some of these studies were published that TREM2 was only expressed in monocytes, hence the staining pattern separate to microglia (7, 12, 51). However, a number of high profile transcriptomic studies have now shown that TREM2 is expressed on DAM/neurodegenerative phenotype of microglia (3, 26, 29). Having said this, TREM2 immunohistochemistry has been shown in other models such as primary cells (19) and mouse models (7, 14). These studies show that TREM2 can be found within myeloid cells surrounding amyloid plaques.

We assessed the levels of expression of a number of genes involved in inflammation and known genes involved in AD pathogenesis. A striking difference was seen between the *TREM2^+^* SAD, *TREM2^−^* SAD and FAD cases compared to the *TREM2^+^* control cases. Visualisation of the canonical pathways and biological functions showed an almost opposite effect between the *TREM2^+^* SAD cases and the *TREM2^+^* control cases whilst, the *TREM2^+^* controls cases showed a downregulation of the majority of the pathways represented. This included neuroinflammatory pathways, predicted to be activated in all AD groups and downregulation only observed in the *TREM2^+^* controls cases. This suggests that *TREM2* variants drive or fuel disease mechanisms once the disease has been initiated. We investigated the protein expression changes in the different groups compared to the controls. There was very little overlap in the top 20 upregulated and downregulated proteins. One overlap was the downregulation of Paxillin (PXN) in both the *TREM2^+^* control cases and *TREM2^+^* SAD cases. PXN is a cytoskeletal protein involved in actin‐membrane attachment at sites of cell adhesion to the extracellular matrix and involved in cell movement and migration (31). This suggests that the microglia cells in the *TREM2^+^* cases are less mobile than *TREM2^−^* cases. This would need further investigation on a homogeneous microglial cell population extracted from postmortem human brain to confirm these findings are associated with the microglia.

We used ingenuity pathway analysis to highlight the top canonical pathways predicted to be activated or inhibited. The top pathways, represented by the proteins identified in the soluble fraction, activated across all disease groups included HIPPO signalling, amyloid processing and neuroinflammation, whereas melatonin signalling, LXR/RXR activation and actin cytoskeleton signalling predicted activation and inhibition were more mixed between groups. The top canonical pathways inhibited across disease groups included EIF2 signalling and glioma signalling. However, like the gene expression analysis the *TREM2^+^* control cases, in the majority of pathways, had the opposite prediction. *TREM2^+^* SAD cases had the highest levels of upregulation of APP protein, *TREM2^+^* controls were the only group to show downregulation of APP. We found in both gene expression and proteomic investigations an increase in GFAP in the *TREM2^+^* SAD cases. However, when we assessed the GFAP pathological load in three different brain regions there were no significant differences between the *TREM2^+^* SAD cases, *TREM2^−^* SAD cases and *TREM2^+^* control cases. This would suggest that different post‐translational modifications in GFAP could be implicated in these differences that are not detected using immunohistochemical techniques.

The main limitation of this study were the number of cases that were available to be studied. Although our results are validated by differences observed here replicating some findings observed in larger studies, none of these studies have looked at the impact *TREM2* variants are having in cases that do not develop Alzheimer’s disease. The novelty of our study is the reporting of the *TREM2^+^* control cases that were cognitively normal and did not have any amyloid plaques or tau pathology present. It has been reported that *TREM2* expression decreases as we age and the brain becomes more vulnerable to neurodegeneration (37). The identification of similar *TREM2^+^* control cases could potentially aid in understanding how *TREM2* variants are involved in disease pathogenesis.

In summary we have shown that *TREM2^+^* SAD cases, with a *R47H* or *D87N* variant, were pathologically indistinguishable from *TREM2^−^* SAD cases, when investigating the Aβ and tau loads. We have shown that *TREM2^+^* control cases with a *R47H* variant, showed no Aβ or tau deposition. All *TREM2^+^* SAD cases carried an *APOE ε4* allele, whereas the *TREM2^+^* control cases did not carry an *APOE ε4* allele. We have previously reported an association between the *TREM2 R47H* variant and the *APOE ε4* allele, which can also be seen throughout the cases identified with *TREM2 R47H* variant in the literature (28, 29, 61). Therefore, the hypothesis that an *APOE ε4* genotype predisposes to the disease and a *TREM2 R47H* variant drives the pathogenesis of AD was introduced (41). Although in a recent paper only 50% of the *TREM2* variant carriers were positive for at least one *APOE ε4* allele, however the authors did not provide details on the specific *TREM2* variant present (46). It will, therefore, be important to analyse the impact of APOE genotypes on specific *TREM2* variants rather than combining the *TREM2* variants into one group. To date there has been no confirmation that different *APOE* alleles bind to TREM2 with differing affinities. However, the *TREM2 R47H* variant has been shown to have reduced ligand binding, including lipoproteins (1). The observation that *TREM2^+^* control cases do not have any AD pathology at time of death, have a strikingly different gene expression and proteomic profile compared to the *TREM2^+^* SAD cases, *TREM2^−^* SAD cases and normal controls, suggests that other factors initiating the disease process are absent.

## Supporting information


**Figure S1.** Top pathways and functions represented from Nanostring data. (A) List of top 30 canonical pathways in Nanostring data listed according to the *z*‐score generated by IPA software. (B) List of top diseases and functions represented in Nanostring data according to *z*‐score given by IPA software. Orange represents a predicted activation of the pathway and blue represents a predicted inhibition of the pathway based on expression values found in the data. Clear differences are observed between *TREM2^+^* SAD, *TREM2^−^* SAD and FAD cases compared to the *TREM2^+^* controls.

**Figure S2.** Canonical pathways and functions represented in proteomic data. (A) Canonical pathways found in the soluble fraction according to *z*‐score given by IPA software, (B) canonical pathways found in the insoluble fraction according to *z*‐score given by IPA software. (C) Diseases and functions found in the soluble fraction according to *z*‐score given by IPA software, (D) diseases and functions found in the insoluble fraction according to *z*‐score given by IPA software. Orange represents a predicted activation of the pathway and blue represents a predicted inhibition of the pathway based on expression values found in the data. Intensity of colour relates to how activated or inhibited the pathway is predicted to be.

**Figure S3**. Canonical pathways represented across nanostring and proteomic data. Canonical pathways found in the nanostring, soluble fraction and insoluble fraction according to *z*‐score given by IPA software. Orange represents a predicted activation of the pathway and blue represents a predicted inhibition of the pathway based on expression values found in the data. Intensity of colour relates to how activated or inhibited the pathway is predicted to be. Each disease group (*TREM2^−^* SAD, *TREM2^+^* SAD and *TREM2^+^* Controls) are outlined by black boxes.

**Table S1.** Significantly altered genes in *TREM2^−^* SAD, *TREM2^+^* SAD, FAD and *TREM2^+^* Controls when compared to neurologically normal controls. Gene ID with an expression change of *P* < 0.05 are listed and shown whether they are upregulated or downregulated.

## Data Availability

The data that support the findings of this study are available from the corresponding author upon reasonable request.

## References

[1] Abduljaleel Z , Al‐Allaf FA , Khan W , Athar M , Shahzad N , Taher MM *et al* (2014) Evidence of Trem2 variant associated with triple risk of alzheimer’s disease. PLoS ONE 9:1–11. 10.1371/journal.pone.0092648 PMC3963925 24663666

[2] Ayer AH , Wojta K , Ramos EM , Dokuru D , Chen JA , Karydas AM *et al* (2019) Frequency of the TREM2 R47H variant in various neurodegenerative disorders. Alzheimer Dis Assoc Disord 33:327–330. 31513029 10.1097/WAD.0000000000000339 PMC7050643

[3] Bisht K , Sharma KP , Lecours C , Sánchez MG , El Hajj H , Milior G *et al* (2016) A new phenotype predominantly associated with pathological states. Glia 64:826–839. 26847266 10.1002/glia.22966 PMC4949554

[4] Boche D , Perry VH , Nicoll JA (2013) Review: activation patterns of microglia and their identification in the human brain. Neuropathol Appl Neurobiol 39:3–18. 23252647 10.1111/nan.12011

[5] Braak Heiko , Braak E (1991) Neuropathological stageing of Alzheimer‐related changes. Acta Neuropathol 82:239–259. 1759558 10.1007/BF00308809

[6] Butovsky O , Jedrychowski MP , Moore CS , Cialic R , Amanda J , Gabriely G *et al* (2014) Identification of a unique TGF‐β dependent molecular and functional signature in microglia. Nat Neurosci 17:131–143. 24316888 10.1038/nn.3599 PMC4066672

[7] Chertoff M , Shrivastava K , Gonzalez B , Acarin L , Giménez‐Llort L (2013) Differential modulation of TREM2 protein during postnatal brain development in mice. PLoS ONE 8:e72083. 23977213 10.1371/journal.pone.0072083 PMC3747061

[8] Coats CJ , Heywood WE , Virasami A , Treibel TA , Moon JC , Ashworth M , *et al*. (2018) Proteomic analysis of the myocardium in hypertrophic obstructive cardiomyopathy. Circ Genom Precis Med 11:e001974. 30562113 10.1161/CIRCGEN.117.001974

[9] Daws MR , Sullam PM , Niemi EC , Chen TT , Tchao NK , Seaman WE (2003) Pattern recognition by TREM‐2: binding of anionic ligands. J Immunol 171:594–599. 12847223 10.4049/jimmunol.171.2.594

[10] Emi M , Wu L , Robertson M , Myers R , Hegele R , Williams R *et al* (1988) Genotyping and sequence analysis of apolipoprotein E isoforms. Genomics 3:373–379. 3243553 10.1016/0888-7543(88)90130-9

[11] Esiri M , Al Izzi M , Reading M (1991) Macrophages, microglial cells, and HLA‐DR antigens in fetal and infant brain. J Clin Pathol 44:102–106. 1864982 10.1136/jcp.44.2.102 PMC496969

[12] Fahrenhold M , Rakic S , Classey J , Brayne C , Ince PG , Nicoll JAR , Boche D (2018) TREM2 expression in the human brain: a marker of monocyte recruitment? Brain Pathol 28:595–602. 28987033 10.1111/bpa.12564 PMC6221091

[13] Forabosco P , Ramasamy A , Trabzuni D , Walker R , Smith C , Bras J *et al* (2013) Neurobiology of Aging Insights into TREM2 biology by network analysis of human brain gene expression data. Neurobiol Aging 34:2699–2714. 23855984 10.1016/j.neurobiolaging.2013.05.001 PMC3988951

[14] Frank S , Burbach GJ , Bonin M , Walter M , Streit W , Bechmann I , Deller T . (2008) TREM2 is upregulated in amyloid plaque‐associated microglia in aged APP23 transgenic mice. Glia 56:1438–1447. 18551625 10.1002/glia.20710

[15] Geiss GK , Bumgarner RE , Birditt B , Dahl T , Dowidar N , Dunaway DL *et al* (2008) Direct multiplexed measurement of gene expression with color‐coded probe pairs. Nat Biotechnol 26:317–325. 18278033 10.1038/nbt1385

[16] Guerreiro R , Ph D , Wojtas A , Bras J , Carrasquillo M , Rogaeva E *et al* (2013) TREM2 variants in Alzheimer’s disease. N Engl J Med 368:117–127. 23150934 10.1056/NEJMoa1211851 PMC3631573

[17] Hickman SE , Kingery ND , Ohsumi TK , Borowsky ML , Wang L , Means TK *et al* (2013) The microglial sensome revealed by direct RNA sequencing. Nat Neurosci 16:1896–1905. 24162652 10.1038/nn.3554 PMC3840123

[18] Hooli BV , Parrado AR , Mullin K , Yip W‐K , Liu T , Roehr JT *et al* (2014) The rare TREM2 R47H variant exerts only a modest effect on Alzheimer disease risk. Am Acad Neurol 83:1353–1358. 10.1212/WNL.0000000000000855 PMC4189101 25186855

[19] Hsieh CL , Koike M , Spusta SC , Niemi EC , Yenari M , Nakamura MC *et al* (2009) A role for TREM2 ligands in the phagocytosis of apoptotic neuronal cells by microglia. J Neurochem 109:1144–1156. 19302484 10.1111/j.1471-4159.2009.06042.x PMC3087597

[20] Jay TR , von Saucken VE , Landreth GE (2017) TREM2 in neurodegenerative diseases. Mol Neurodegener 12. 10.1186/s13024-017-0197-5. PMC5541421 28768545

[21] Jiang T , Tan L , Zhu X , Zhang Q , Cao L , Tan M *et al* (2014) Upregulation of TREM2 ameliorates neuropathology and rescues spatial cognitive impairment in a transgenic mouse model of Alzheimer’s disease. Neuropsychopharmacology 39:2949–2962. 25047746 10.1038/npp.2014.164 PMC4229581

[22] Jiang T , Tan L , Zhu X , Zhou J , Cao L , Tan M *et al* (2015) Neurobiology of aging silencing of TREM2 exacerbates tau pathology, neurodegenerative changes, and spatial learning de fi cits in P301S tau transgenic mice. Neurobiol Aging 36:3176–3186. 26364736 10.1016/j.neurobiolaging.2015.08.019

[23] Jiang T , Zhang Y , Chen Q , Gao Q , Zhu X (2016) Neuropharmacology TREM2 modi fi es microglial phenotype and provides neuroprotection in P301S tau transgenic mice. Neuropharmacology 105:196–206. 26802771 10.1016/j.neuropharm.2016.01.028

[24] Jones BM , Bhattacharjee S , Dua P , Hill JM , Zhao Y , Lukiw W (2014) Regulating amyloidogenesis through the natural triggering receptor expressed in myeloid/microglial cells 2 (TREM2). Front Cell Neurosci 8:1–3. 24744699 10.3389/fncel.2014.00094 PMC3978349

[25] Jonsson T , Stefansson H , Steinberg S , Jonsdottir I , Jonsson PV , Snaedal J *et al* (2013) Variant of TREM2 associated with the risk of AD. N Engl J Med 368:107–116. 23150908 10.1056/NEJMoa1211103 PMC3677583

[26] Keren‐Shaul H , Spinrad A , Weiner A , Matcovitch‐Natan O , Dvir‐Szternfeld R , Ulland TK *et al* (2017) A unique microglia type associated with restricting development of Alzheimer’s disease. Cell 169:1276–1290.e17. 28602351 10.1016/j.cell.2017.05.018

[27] Kober D , Wanhainen K , Johnson B , Randolph D , Holtzman M , Brett T (2014) Preparation, crystallization, and preliminary crystallographic analysis of wild‐type and mutant human TREM‐2 ectodomains linked to neurodegenerative and inflammatory diseases. Protein Expr Purif 96:32–38. 24508568 10.1016/j.pep.2014.01.015 PMC3980731

[28] Korvatska O , Leverenz JB , Jayadev S , McMillan P , Kurtz I , Guo X *et al* (2015) R47H variant of *TREM2* associated with alzheimer disease in a large late‐onset family. JAMA Neurol 72:920. 26076170 10.1001/jamaneurol.2015.0979 PMC4825672

[29] Krasemann S , Madore C , Cialic R , Baufeld C , Calcagno N , El Fatimy R *et al* (2017) The TREM2‐APOE pathway drives the transcriptional phenotype of dysfunctional microglia in neurodegenerative diseases. Immunity 47:566–581.e9. 28930663 10.1016/j.immuni.2017.08.008 PMC5719893

[30] Lashley T , Rohrer JD , Bandopadhyay R , Fry C , Ahmed Z , Isaacs AM *et al* (2011) A comparative clinical, pathological, biochemical and genetic study of fused in sarcoma proteinopathies. Brain 134:2548–2564. 21752791 10.1093/brain/awr160 PMC3170529

[31] López‐colomé AM , Lee‐rivera I , Benavides‐hidalgo R , López E (2017) Paxillin: a crossroad in pathological cell migration. J Hematol Oncol 10:1–15. 28214467 10.1186/s13045-017-0418-y PMC5316197

[32] Lue L , Schmitz CT , Sorrano G , Sue LI , Beach TG , Walker DG (2015) TREM2 protein expression changes correlate with Alzheimer’s disease neurodegenerative pathologies in postmortem temporal cortices. Brain Pathol 25:469–480. 25186950 10.1111/bpa.12190 PMC4427527

[33] Malkki H (2015) Alzheimer disease: the involvement of TREM2 R47H variant in Alzheimer disease confirmed, but mechanisms remain elusive. Nat Rev Neurol Gr 11:307. 10.1038/nrneurol.2015.84 25986503

[34] Martiskainen H , Viswanathan J , Nykänen N (2015) Neurobiology of Aging Transcriptomics and mechanistic elucidation of Alzheimer’s disease risk genes in the brain and in vitro models. Neurobiol Aging 36:1221.e15–1221.e28. 10.1016/j.neurobiolaging.2014.09.003 25281018

[35] Matarin M , Salih DA , Hardy J , Edwards FA , Matarin M , Salih DA *et al* (2015) A genome‐wide gene‐expression analysis and database in transgenic mice during development of amyloid or tau pathology resource a genome‐wide gene‐expression analysis and database in transgenic mice during development of amyloid or tau pathology. Cell Rep 10:633–644. 25620700 10.1016/j.celrep.2014.12.041

[36] Mattiace LA , Davies P , Dennis W (1990) Detection of HLA‐DR on microglia in the human brain is a function of both clinical and technical factors. Am J Pathol 136:1101–1115. 1693471 PMC1877424

[37] Mecca C , Giambanco I , Donato R , Arcuri C (2018) Microglia and aging: the role of the TREM2–DAP12 and CX3CL1‐CX3CR1 Axes. Int J Mol Sci 19:1–27. 10.3390/ijms19010318 PMC5796261 29361745

[38] Melchior B , Garcia AE , Hsiung B‐K , Lo KM , Doose JM , Thrash JC *et al* (2010) Dual induction of TREM2 and tolerance‐related transcript, Tmem176b, in amyloid transgenic mice: implications for vaccine‐based therapies for Alzheimer’s disease. ASN Neuro 2:AN20100010. 10.1042/AN20100010 PMC2905103 20640189

[39] Montine T , Phelps C , Beach T , Bigio E , Cairns N , Dickson D *et al* (2012) National Institute on Aging‐Alzheimer’s Association guidelines for the neuropathologic assessment of Alzheimer’s disease: a practical approach. Acta Neuropathol 123:1–11. 22101365 10.1007/s00401-011-0910-3 PMC3268003

[40] Murray CE , Gami‐patel P , Gkanatsiou E , Brinkmalm G , Portelius E , Wirths O *et al* (2018) The presubiculum is preserved from neurodegenerative changes in Alzheimer’s disease. Acta Neuropathol Commun 6:1–17. 30029687 10.1186/s40478-018-0563-8 PMC6053705

[41] Murray C , King A , Troakes C , Hodges A , Lashley T (2019) APOEe4 is also required in TREM2R47H variant carriers for Alzheimer’s disease to develop In. Neuropathol Appl Neurobiol 45:183–186. 29411406 10.1111/nan.12474

[42] Neumann H , Daly MJ (2013) Variant TREM2 as risk factor for Alzheimer’s disease. N Engl J Med 368:182–184. 23151315 10.1056/NEJMe1213157

[43] Ortega‐cubero S , Lorenzo‐betancor O , Lorenzo E , Agundez JA , Jimenez‐Jimenez F , Ross OA *et al* (2015) TREM2 R47H variant and risk of essential tremor: A cross‐sectional international multicenter study. Park Relat Disord 21:306–309. 10.1016/j.parkreldis.2014.12.010 PMC4408541 25585992

[44] Paradowska‐gorycka A , Jurkowska M (2013) Structure, expression pattern and biological activity of molecular complex TREM‐2 / DAP12. Hum Immunol 74:730–737. 23459077 10.1016/j.humimm.2013.02.003

[45] Park M , Yi J , Kim E , Yoon I , Lee E , Lee H *et al* (2015) Triggering receptor expressed on myeloid cells 2 (TREM2) promotes adipogenesis and diet‐induced obesity. Diabetes 64:117–127. 25114293 10.2337/db13-1869

[46] Prokop S , Miller KR , Labra SR , Pitkin RM , Sneha H , Changolkar L *et al* (2019) Impact of TREM2 risk variants on brain region‐specific immune activation and plaque microenvironment in Alzheimer ’ s disease patient brain samples. Acta Neuropathol 138:613–630. 31350575 10.1007/s00401-019-02048-2 PMC6939638

[47] Raha‐Chowdhury R , Henderson JW , Raha AA , Stott SRW , Vuono R , Foscarin S *et al* (2018) Erythromyeloid‐Derived TREM2: A major determinant of Alzheimer’s disease pathology in down syndrome. J Alzheimer’s Dis 61:1143–1162. 29278889 10.3233/JAD-170814 PMC5817909

[48] Raha‐Chowdhury R , Henderson J , Raha A , Vuono R , Bickerton A , Jones E *et al* (2019) Choroid plexus acts as gatekeeper for TREM2, abnormal accumulation of ApoE, and fibrillary tau in Alzheimer’s disease and in down syndrome dementia. J Alzheimer’s Dis 69:91–109. 30909239 10.3233/JAD-181179 PMC6598012

[49] Rosenthal S , Bamne M , Wang X , Berman S , Snita B , Klunk WE *et al* (2015) More evidence for association of a rare TREM2 mutation (R47H) with Alzheimer’s disease risk. Neurobiol Aging 36:1–13. 26058841 10.1016/j.neurobiolaging.2015.04.012 PMC4465085

[50] Roses AD . (1996) Apolipoprotein e alleles as risk factors in alzheimer's disease. Annu Rev Med 47:387–400. 8712790 10.1146/annurev.med.47.1.387

[51] Satoh J , Kawana N , Yamamoto Y , Ishida T , Saito Y , Arima K (2013) A survey of TREM2 antibodies reveals neuronal but not microglial staining in formalin‐fixed paraffin‐embedded postmortem Alzheimer’s brain tissues. Alzheimer’s Res Ther 5:4–6. 23830013 10.1186/alzrt184 PMC3978710

[52] Slattery C , Beck J , Harper L , Adamson G , Abdi Z , Uphill J *et al* (2014) Trem2 variants increase risk of typical early‐onset Alzheimer’s disease but not of prion or frontotemporal dementia. J Neurol Neurosurg Psychiatry 85:e3. 10.1016/j.jalz.2014.05.1751 PMC4627504 25160042

[53] Streit WJ , Braak H , Xue Q‐S , Bechmann I (2009) Dystrophic (senescent) rather than activated microglial cells are associated with tau pathology and likely precede neurodegeneration in Alzheimer’s disease. Acta Neuropathol 118:475–485. 19513731 10.1007/s00401-009-0556-6 PMC2737117

[54] Takahashi K , Rochford CDP , Neumann H (2005) Clearance of apoptotic neurons without inflammation by microglial triggering receptor expressed on myeloid cells‐2. J Exp Med 201:647–657. 15728241 10.1084/jem.20041611 PMC2213053

[55] Thal DR , Rüb U , Orantes M , Braak H (2002) Phases of Aβ‐deposition in the human brain and its relevance for the development of AD. Neurology 58:1791–1800. 12084879 10.1212/wnl.58.12.1791

[56] Rohn TT (2013) The triggering receptor expressed on myeloid cells 2: “trem‐ming” the inflammatory component associated with Alzheimer’s disease. Oxid Med Cell Longev 2013:860959. 23533697 10.1155/2013/860959 PMC3606781

[57] Varvel NH , Grathwohl SA , Degenhardt K , Resch C , Bosch A , Jucker M *et al* (2015) Replacement of brain‐resident myeloid cells does not alter cerebral amyloid‐β deposition in mouse models of Alzheimer ’ s disease. J Exp Med 212:1803–1809. 26458770 10.1084/jem.20150478 PMC4612086

[58] Wang Y , Cella M , Mallinson K , Ulrich JD , Katherine L , Robinette ML *et al* (2015) TREM2 lipid sensing sustains microglia response in an Alzheimer’s disease model. Cell 160:1061–1071. 25728668 10.1016/j.cell.2015.01.049 PMC4477963

[59] Wang Y , Ulland TK , Ulrich JD , Song W , Tzaferis JA , Hole JT *et al* (2016) TREM2‐mediated early microglial response limits diffusion and toxicity of amyloid plaques. J Exp Med 213:667–675. 27091843 10.1084/jem.20151948 PMC4854736

[60] Xiang X , Piers TM , Wefers B , Zhu K , Mallach A , Brunner B *et al* (2018) The Trem2 R47H Alzheimer’s risk variant impairs splicing and reduces Trem2 mRNA and protein in mice but not in humans. Mol Neurodegener 13:1–14. 30185230 10.1186/s13024-018-0280-6 PMC6126019

[61] Yuan P , Condello C , Keene CD , Wang Y , Bird TD , Paul SM *et al* (2016) TREM2 Haplodeficiency in Mice and Humans Impairs the Microglia Barrier Function Leading to Decreased Amyloid Compaction and Severe Axonal Dystrophy. Neuron 92:252–264. 27710785 10.1016/j.neuron.2016.09.016

[62] Zhang B , Kirov S , Snoddy J (2005) WebGestalt: An integrated system for exploring gene sets in various biological contexts. Nucleic Acids Res 33(Suppl. 2):741–748. 10.1093/nar/gki475 PMC1160236 15980575

[63] Zhou Y , Song WM , Andhey PS , Swain A , Levy T , Miller KR *et al* (2020) Human and mouse single‐nucleus transcriptomics reveal TREM2‐dependent and TREM2‐independent cellular responses in Alzheimer’s disease. Nat Med 26:131–442. 31932797 10.1038/s41591-019-0695-9 PMC6980793

