# Supplementary figures and images for: Investigation of pathology, expression and proteomic profiles in human TREM2 variant postmortem brains with and without Alzheimer’s disease
Source: Brain Pathol. 2020 Apr 29;30(4):794–810. doi: 10.1111/bpa.12842 (PMC8018003; doi:10.1111/bpa.12842)

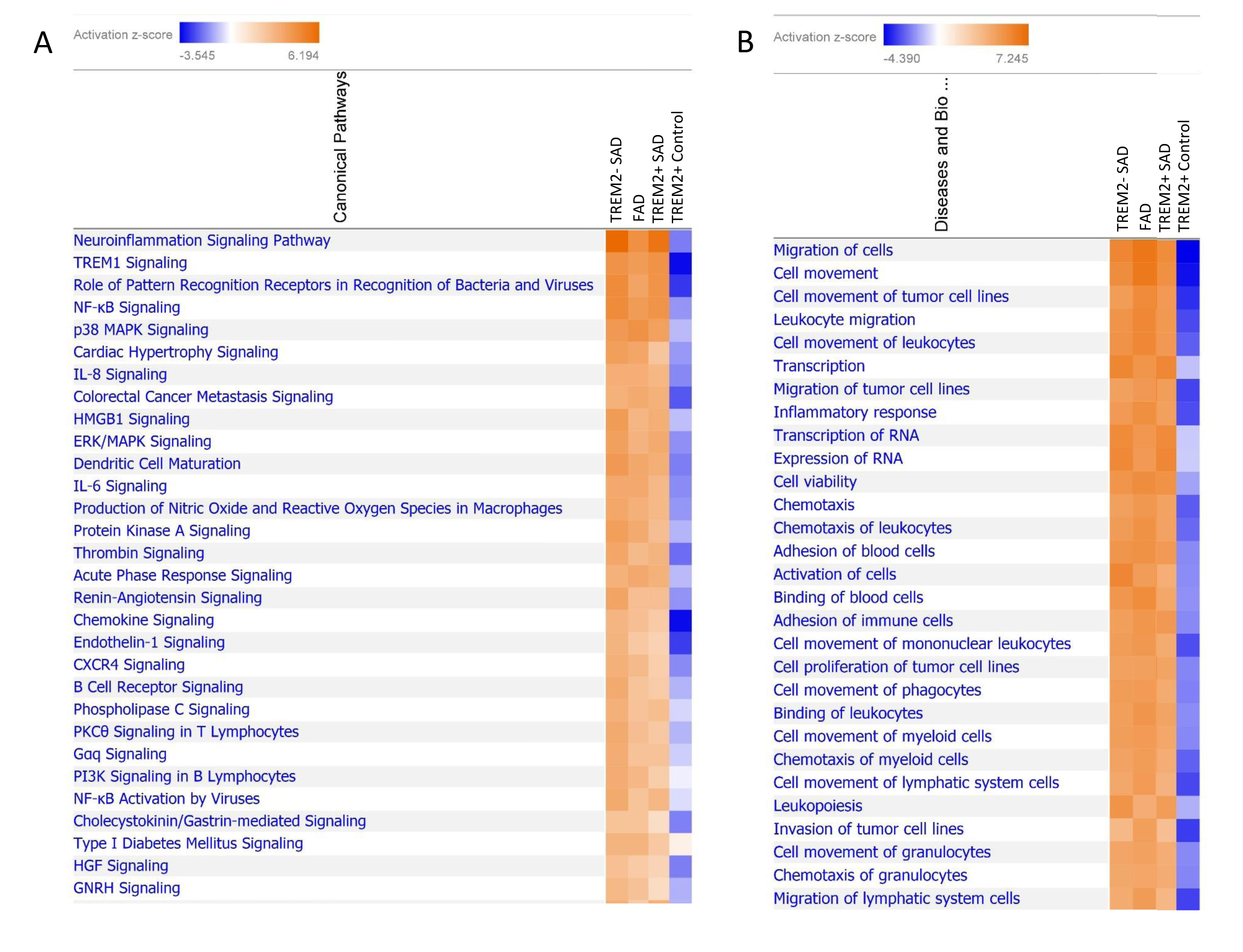

Supplement: Supplementary file 1 — Figure S1. Top pathways and functions represented from Nanostring data. (A) List of top 30 canonical pathways in Nanostring data listed according to the z‐score generated by IPA software. (B) List of top diseases and functions represented in Nanostring data according to z‐score given by IPA software. Orange represents a predicted activation of the pathway and blue represents a predicted inhibition of the pathway based on expression values found in the data. Clear differences are observed between TREM2+ SAD, TREM2− SAD and FAD cases compared to the TREM2+ controls. [file BPA-30-794-s003.tif]

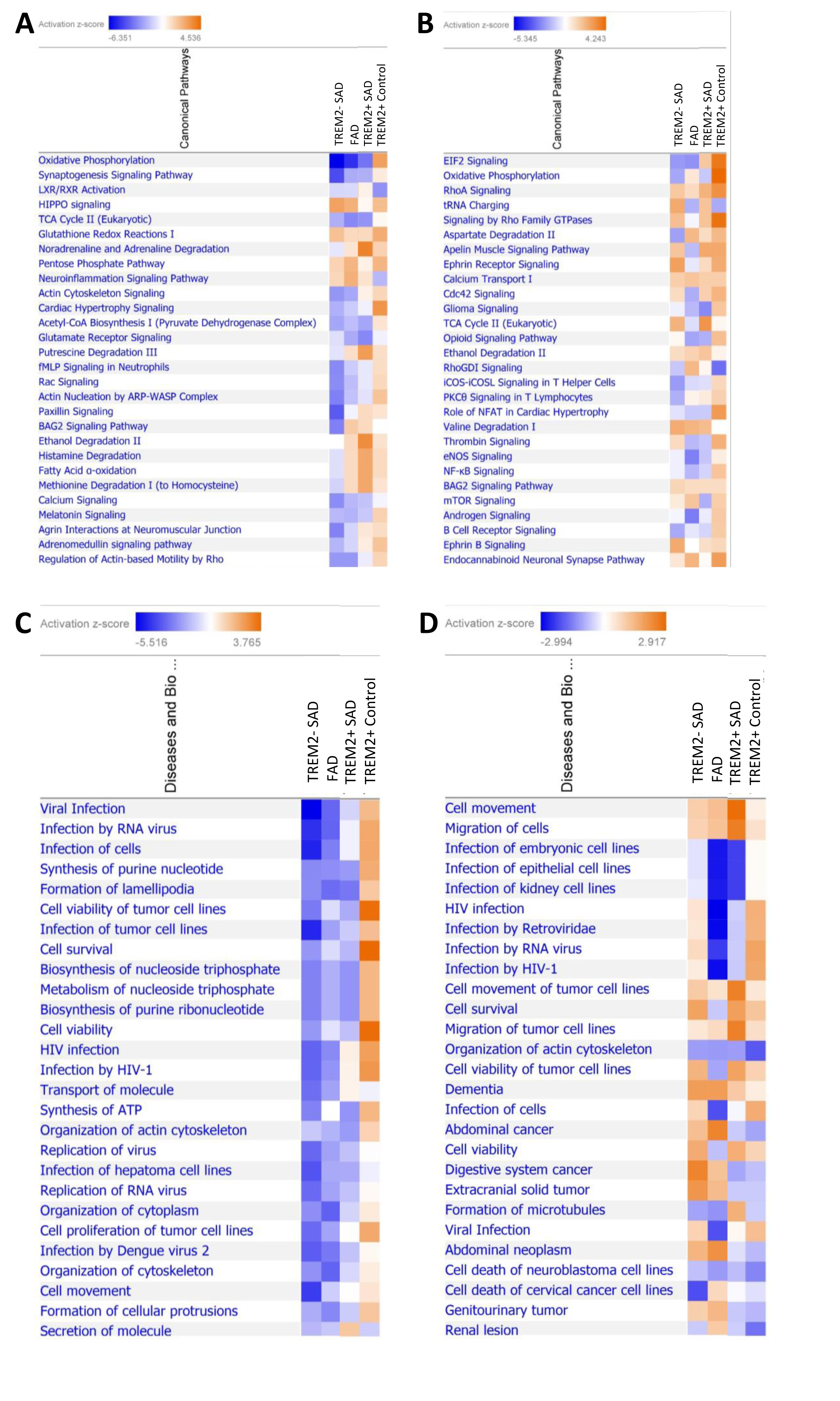

Supplement: Supplementary file 2 — Figure S2. Canonical pathways and functions represented in proteomic data. (A) Canonical pathways found in the soluble fraction according to z‐score given by IPA software, (B) canonical pathways found in the insoluble fraction according to z‐score given by IPA software. (C) Diseases and functions found in the soluble fraction according to z‐score given by IPA software, (D) diseases and functions found in the insoluble fraction according to z‐score given by IPA software. Orange represents a predicted activation of the pathway and blue represents a predicted inhibition of the pathway based on expression values found in the data. Intensity of colour relates to how activated or inhibited the pathway is predicted to be. [file BPA-30-794-s002.tif]

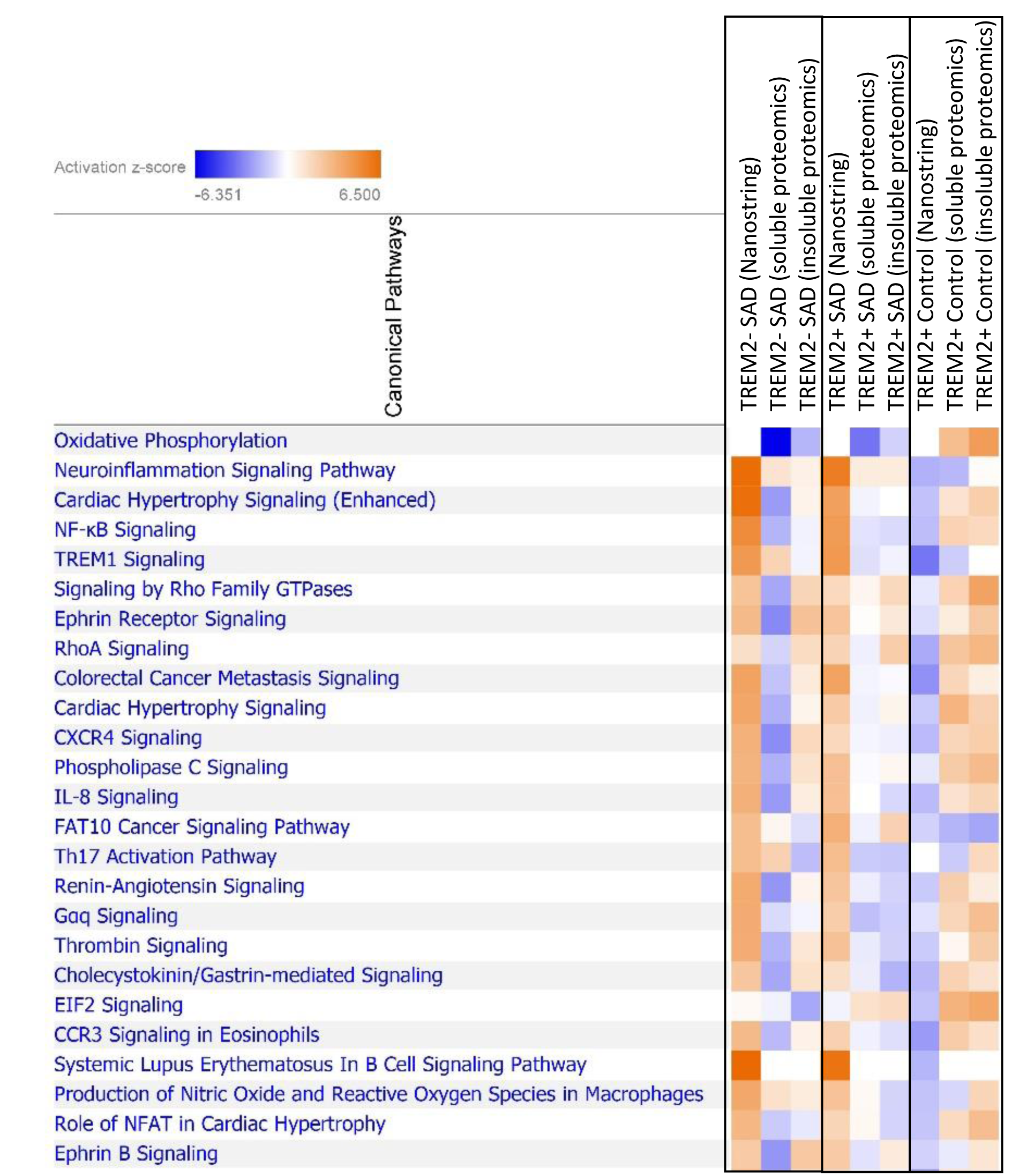

Supplement: Supplementary file 3 — Figure S3. Canonical pathways represented across nanostring and proteomic data. Canonical pathways found in the nanostring, soluble fraction and insoluble fraction according to z‐score given by IPA software. Orange represents a predicted activation of the pathway and blue represents a predicted inhibition of the pathway based on expression values found in the data. Intensity of colour relates to how activated or inhibited the pathway is predicted to be. Each disease group (TREM2− SAD, TREM2+ SAD and TREM2+ Controls) are outlined by black boxes. [file BPA-30-794-s004.tif]
